# Outcome of endoscopic trans-ethmosphenoid optic canal decompression for indirect traumatic optic neuropathy in children

**DOI:** 10.1186/s12886-018-0792-4

**Published:** 2018-06-26

**Authors:** Bo Yu, Yingbai Chen, Yingjie Ma, Yunhai Tu, Wencan Wu

**Affiliations:** 1 Department of Orbital and Oculoplastic Surgery, Eye Hospital of Wenzhou Medical University, No.270 West Xueyuan Road, Wenzhou, Zhejiang, People’s Republic of China; 2 Department of Ophthalmology, Shanghai Jiao Tong University School of Medicine Affiliated Renji Hospital, Shanghai, China; 3 Eye Hospital of Wenzhou Medical University, Wenzhou, Zhejiang, China

**Keywords:** Trans-ethmosphenoid optic canal decompression, Indirect traumatic optic neuropathy, Children

## Abstract

**Background:**

To evaluate the safety and outcomes of endoscopic trans-ethmosphenoid optic canal decompression (ETOCD) for children with indirect traumatic optic neuropathy (ITON).

**Methods:**

From July 1st, 2008 to July 1st, 2015, 62 children diagnosed with ITON who underwent ETOCD were reviewed. Main outcome measure was improvement in visual acuity after treatment.

**Results:**

Altogether 62 children (62 eyes) with a mean age of 11.26 ± 4.14 years were included. Thirty-three (53.2%) of them had residual vision before surgery while 29 (46.8%) had no light perception (NLP). The overall visual acuity improvement rate after surgery was 54.84%. The improvement rate of patients with residual vision (69.70%) was significant higher than that of patients with no light perception (NLP) (37.9%) (*P* = 0.012). However, no significant difference was shown among patients with different residual vision (*P* = 0.630). Presence of orbital and/ or optic canal fracture and hemorrhage within the post-ethmoid and/or sphenoid sinus resulted in poor postoperative visual acuity, duration of presenting complaints did not affect final visual acuity or did not effect outcomes. Intervention performed in children presenting even after 7 days from the injury did not influence the final visual outcome. Three patients developed cerebrospinal fluid rhinorrhea and one encountered cavernous sinus hemorrhage during surgery. No other severe complications were observed.

**Conclusion:**

Children with residual vision had better postoperative visual prognosis and benefited more from ETOCD than children with NLP. Intervention performed in children presenting even after 7 days from the injury did not influence the final visual outcome, however, this needs to be reassessed in children presenting long after the injury.Treatment should still be recommended even for cases of delayed presentation to hospital.

## Background

The most common cause of unilateral blindness for children is trauma [1]. Traumatic optic neuropathy (TON) is a relatively rare but potentially severe complication of head injury. Affecting about 5% of patients with closed head injuries, TON often causes devastating permanent visual loss [2].TON can be caused by direct or indirect injury. Direct optic injury always brings poor prognosis because of optic nerve avulsion or laceration, or direct fracture of the optic canal. Indirect traumatic optic neuropathy (ITON) is caused by increased intracanalicular pressure after an injury, which usually initiates a cascade of molecular and chemical mediators. Secondary disorders, such as intraneural edema, hematoma, altered microvasculature or cerebrospinal fluid circulation, and interruption of direct axoplasmic transport, will happened due to these changes [3]. These conditions are partially reversible and may get benefit from medicine or surgical treatment [4].

The treatment protocol of ITON is relatively well defined in adult, suggesting endoscopic trans-ethmosphenoid optic canal decompression (ETOCD) is feasible way to improve outcome [2, 4, 5]. However, its application to children is far from agreement, with only a few studies available. In this case series, we set out to explore the outcome of ETOCD for children with ITON.

## Methods

### Clinical data

From July 1st, 2008 to July 1st, 2015, 62 pediatric cases with unilateral ITON were indentified from the electronic medical record system of Eye Hospital of Wenzhou Medical University. ITON was diagnosed based on severe reduction or loss in visual acuity (VA) accompanied by a relative afferent pupillary defect (RAPD) after a closed head injury. If necessary, visual field examination and visual evoked potential (VEP) were performed for diagnosis. High-resolution computed tomography (HRCT) scan of the head and the orbit was performed in all the patients. All cases after admission were administered by methylprednisolone (20 mg/kg/day) and mouse-derived nerve growth factor (NGF) (30 μg/ml/day) (Staidson (Beijing) Biopharmaceuticals Co. Ltd) for 3 days. Patients with no VA improvement after intravenous treatment were recommended to endoscopic trans-ethmosphenoid optic canal decompression (ETOCD) and followed up to 3 months after surgery. There were 29 patients who didn’t subjected to surgery. Twenty-one of them showed VA improvement after 3 days of methylprednisolone and NGF and 8 of them refused surgery even with no VA improvement after medicine treatment. Children with bilateral ITON were also not included because these patients were accompanied by cranial cerebral injury or optic chiasm injury, making the cranial surgery priority. Written consent was obtained from all children’s parents or guardians before surgery in accordance with institutional review board’s policies. The research followed the principles of the Declaration of Helsinki. This research was approved by ethics committees of Eye Hospital of Wenzhou Medical University.

We recorded the demographic characteristics of the patients, VA before surgery, VA at the 3 months after surgery, time to medical treatment, time of complaint of visual loss, together with other accompanied conditions like consciousness impairment, hemorrhage within the post-ethmoid and/or sphenoid sinus, orbital fracture and optic canal fracture.VA improvement is defined as (a) VA improved ≥2 lines on the Snellen visual chart, (b) VA improved from no light perception to light perception or better, (c) VA improved from light perception to hand motion or better, (d) VA improved from hand motion to finger counting or better [5, 6].

### Surgical procedure

The choice of ETOCD in this study was chosen based on pre-determined clinical data.

**Fig. 1:**
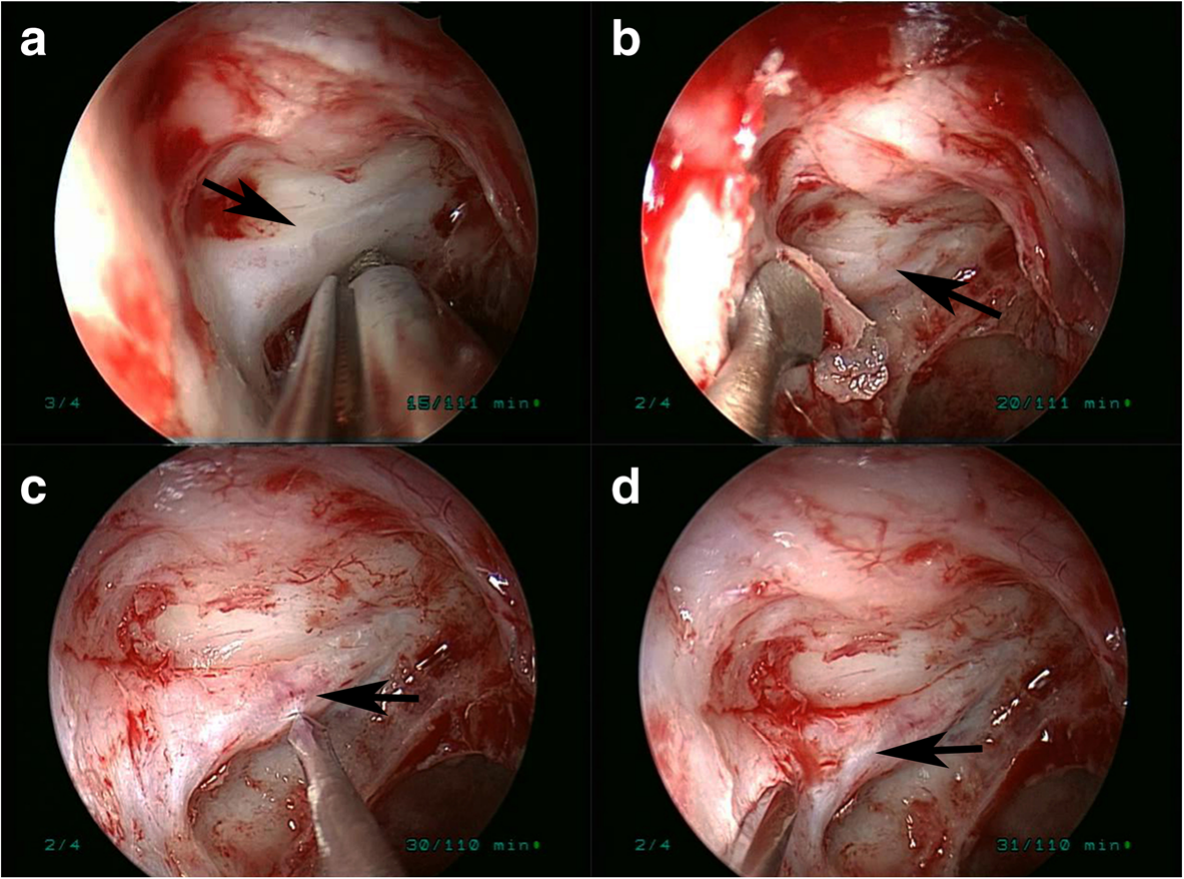
The surgical procedures of ETOCD. **a** The medial wall of optic canal was thinned by a microdrill. **b** The thinned medial wall of optic canal was removed by a micro-curette. **c** The periorbita of the orbital apex and annulus of Zinn was split wtih a sharp 9# MVR knife. **d** The optic nerve sheath was incised to create small intermittented punctuation. (The black arrow indicates the optic canal)

All of the procedures of ETOCD were performed under general anesthesia by a single orbital surgeon (Dr. WW). A routine endoscopic sphenoethmoidectomy was performed using the Messerklinger technique with preservation of the middle turbinate. The sphenoid face is opened widely and the bulge of the optic nerve canal is identified along the lateral wall of the sphenoid sinus, superior to the carotid artery. In some patients, the optic canal may be identified initially in a posterior ethmoid or Onodi cell, which can be identified on preoperative CT scan. Identification and opening of the Onodi cell provides adequate surgical exposure and allow full access to the optic canal. For some children whose the sphenoid sinus was not fully developed, a navigation system were used during surgery to locate the optic nerve canal. After sphenoethmoidectomy, the lesser wing of the sphenoidal bone and the medial wall of the optic canal were thinned with a microdrill and removed with a micro-curette. Then, the periorbita of the orbital apex, annulus of Zinn and the optic nerve sheath were incised with a sharp 9# MVR knife. Finally, the operating field was covered by a piece of sterilized gelatin sponge immersed with dexamethasone (5 mg/2 ml) and mouse-derived NGF (30 μg/ml). Postoperative administration of methylprednisolone (20 mg/kg/day) was given for 4 days, ceftriaxone (2.0 g/day) for 5 days and NGF (30 μg/ml /day) for 1 month (Fig. 1).

### Statistical analysis

Statistical analyses were performed with SPSS 17.0 software (SPSS Inc., Chicago, IL). Multiple linear-regression analysis was performed to identify independent predictors for postoperative VA improvement in patients. Univariate analysis was performed by chi square test and Fisher exact test to compare the postoperative visual improvement rate among different preoperative VA groups. Results were considered significant at P<0.05.

## Results

Among the included 62 children (62 eyes), 53 were boys and 9 were girls. The age ranged from 3 to 17 years with a mean age of 11.26 ± 4.14 years. The most common cause of ITON in our study was fall (29/62), followed by car accident (22/62) and assaults (11/62). There were 26 patients who experienced consciousness impairment after trauma. Orbital bone fracture was found in 31 patients and hemorrhage within the post-ethmoid and/or sphenoid sinus was found in 22 patients. Optic canal fracture was shown on preoperative CT scan in 9 cases and detected during surgery in another 18 cases. Only 13 children were brought to our hospital for treatment within 3 days after trauma and 13 children were treated from day 3 to day 7. Another 36 children got treatment later than 7 days after trauma. The mean duration from injury for the patients treated > 7 days was 17.1 days (ranging from 9 to 29 days).41 patients complained of visual loss immediately after the trauma. Of all the 62 patients, 33 patients had residual vision before surgery and 29 patients had no light perception. There were only 10 patients with preoperative VA better than 0.1. (Table 1).

**Table 1:** Clinical characteristic of 62 Children with TON

Characteristic	Case (n) (%)
Injury type	
Fall	29 (46.8)
Car accident	22 (35.5)
Assault	11 (17.7)
State of consciousness	
Impairment	26 (41.9)
None	36 (58.1)
Orbital bone fracture	
Yes	31 (50.0)
No	31 (50.0)
Hemorrhage within the post-ethmoid and/or sphenoid sinus	
Yes	22 (35.5)
No	40 (64.5)
Optic canal fracture	
On image scan	9 (14.5)
Found during surgery	27 (43.5)
None	35 (56.5)
Time to medical treatment	
Within 3 days	13 (21.0)
Between 3 and 7 days	13 (21.0)
Later than 7 days	36 (58.0)
Time of visual loss development after injury	
Immediate	41 (66.1)
Secondary	21 (33.9)
Baseline Visual acuity	
NLP	29 (46.8)
LP	7 (11.3)
HM to 20/200	16 (25.8)
≥ 20/200	10 (16.1)

*NLP* no light perception, *LP* light perception, *HM* hand movement

The multiple linear-regression analysis showed that the existence of orbital fracture, optic canal fracture and hemorrhage within the post-ethmoid and/or sphenoid sinus were the independent predictors for poor postoperative visual acuity. The baseline visual acuity was also an independent predictor. On the contrary, age, conscious status, time to medical treatment and time of visual loss development after injury did not independent influence the final BCVA. (Table 2).

**Table 2:** Multiple linear regression to predict postoperative visual improvement

Variable	Coefficient	SE	t	P
Age	−0.004	0.014	−0.261	0.795
Consciousness impairment	0.045	0.115	0.393	0.696
Orbital fracture	−0.250	0.114	−2.205	0.032*
Optic canal fracture	−0.275	0.120	−2.292	0.026*
Hemorrhage within the post-ethmoid and/or sphenoid sinus	−0.266	0.123	−2.168	0.035*
Time to medical treatment	−0.008	0.008	−0.972	0.336
Time of visual loss development after injury	0.067	0.121	0.550	0.585
Baseline visual acuity	0.124	0.056	2.197	0.032*

*indicate independent predictor for visual improvement (p<0.05)

Postoperative VA improvement was achieved in 34 patients, suggesting an overall effective rate of 54.84% (34/62). For patients with NLP before surgery, 11(37.9%) of them gained VA improvement after surgery while 18 (62.1%) of them didn’t. For patients with residual vision before surgery, 23 (69.70%) had visual improvement and 9 (27.27%) of them didn’t. The improvement rate of patients with preoperative residual vision is significant higher than that of patients with NLP (*P* = 0.012). However, no significant difference was observed among patients with different preoperative residual vision (*P* = 0.630) (Table 3).

**Table 3:** Comparison of surgical effect of patients with different preoperative visual acuity

Visual acuity before surgery	Visual acuity after surgery				Improved (n)	Same (n)	Reduced (n)	Improvement Rate (%)
	NLP	LP	HM to 20/200	≥20/200				
NLP	18	2	4	5	11	18	0	37.93
Non-NLP					23	9	1	69.70^a^
LP	0	3	4	0	4	3	0	57.14
HM to 20/200	0	0	7	9	11	4	1	68.75
≥20/200	0	0	0	10	8	2	0	80.00

*NLP* no light perception, *LP* light perception, *HM* hand movement

^a^Non-NLP group has higher effective rate as compared with NLP group (χ^2^ = 6.289 *P* = 0.012)

During surgery, 3 patients developed cerebrospinal fluid rhinorrhea (CSFR). Two of them were repaired by mucosal flap transplantation uneventfully during surgery and one recovered from strict bed rest in a 30 degree head-up position. One patient experienced cavernous sinus hemorrhage during surgery and the hemorrhage was controlled by compression. No other severe complications were observed.

## Discussion

Treatment for ITON among children remains controversy and no definite conclusion has been reached yet. According to previous studies, the VA improvement rate of high-dose steroid treatment, optic canal decompression (OCD) and high-dose steroid combined with OCD were 36.36–40%, 21.42–71% and 53.84–82.92% respectively [1, 7–10]. OCD with or without combination of steroid is generally thought to be more effective in visual improvement than steroid treatment only [7, 8, 10]. OCD physically decompresses the nerve within the canal, thereby creating the space for the nerve to swell, limiting the damaging effect of compression and helping the re-establishment of optic nerve function [2, 9, 11]. Endoscopic approach of OCD is gaining its preference for its better surgical visualization, reduced hospitalization and less morbidity as compared with intracranial approaches [4]. In this research, we reported an overall effective rate of 54.84% after ETOCD, 37.93% for children with NLP before surgery and 69.70% for children with preoperative residual vision.

In this study, we find out that orbital fracture, hemorrhage within the post-ethmoid and/or sphenoid sinus and optic canal fracture (OCF) are independent factors for VA prognosis. This is consistent with previous findings among adults [12–17]. The existence of orbital fractures is usually associated with large external forces, which means severe optic nerve damage. Hemorrhage within the post-ethmoid and/or sphenoid sinus is closely associated with great energy upon craniofacial region [14, 18] and may induce toxic substances which aggravate optic nerve damage [15, 19]. OCF may cause devastating damage to optic nerve by bony fragment injury and/or structural deformation, followed by increased intracanalicular pressure or even intracanalicular hematoma [13]. Therefore, surgical intervention is advocated once OCF is identified [20, 21].

Preoperative VA is also an independent predictive for postoperative VA improvement. VA improvement rate is 37.93% for patients with NLP and 69.70% for patients with residual vision, which shows significant difference (*P* = 0.012). However, no significant difference is found among patients with different preoperative residual vision. It is indicated that the presence of preoperative residual vision is crucial for VA prognosis and patients with eyesight left may benefit more from the surgical intervention. Absence of residual vision implies the probable degeneration and apoptosis of ganglionic cells of the optic nerve [22].

Research among adults showed that patients receiving early treatment (equal or less than 7 days) have better VA prognosis [16, 23]. On the contrary, no correlation is detected between the time to medical treatment and VA prognosis among children in this study. Previous study reported that children are less likely to develop long-term chronic neuropathic pain syndromes than adults following nerve injury and the overall effective rate of OCD in pediatric cases is remarkably higher than the adults [24]. This lead us to make assumption that children may have better potential and ability to rehabilitation. Another possible explanation is the anatomical difference. The sphenoid turbinate is not close to the corpus sphenoidale until they are about 3 years old.Till then, the sphenoid turbinate cavity starts to develop into sphenoid sinus cavity. Normally, the sphenoid sinus does not begin to pneumatize until they are about 6 years old, and the pneumatization normally accomplished when they are about 10 years old. Thus significant differences are found in size, form, pneumatization degree, and the septum of the sphenoid sinus among children [2]. Thus, the force and the extent of optic nerve damage after ITON are different between adults and pediatric case. This reminds us that we should never give up pediatric cases too early even when they are delayed for treatment.

ETOCD for children is believed to be much more difficult than for adults. Certain important anatomical structures of children have not fully developed yet, such as sinuses, skull base, orbits, and optic canals [1, 2, 9]. In our study, 8 of 62 patients (12.9%) had undeveloped sphenoid sinus which make it difficult to identify the optic nerve canal bulge. The narrow nasal cavity largely restricts the flexibility of the endoscope and other surgical instruments. In addition, the adhesion between the optic canal and optic sheath is much tighter and the bone of optic nerve canal is more fragile, which requires extremely delicate operation while grinding the the bony structure of optic canal and separating it from the optic nerve sheath.

**Fig. 2:**
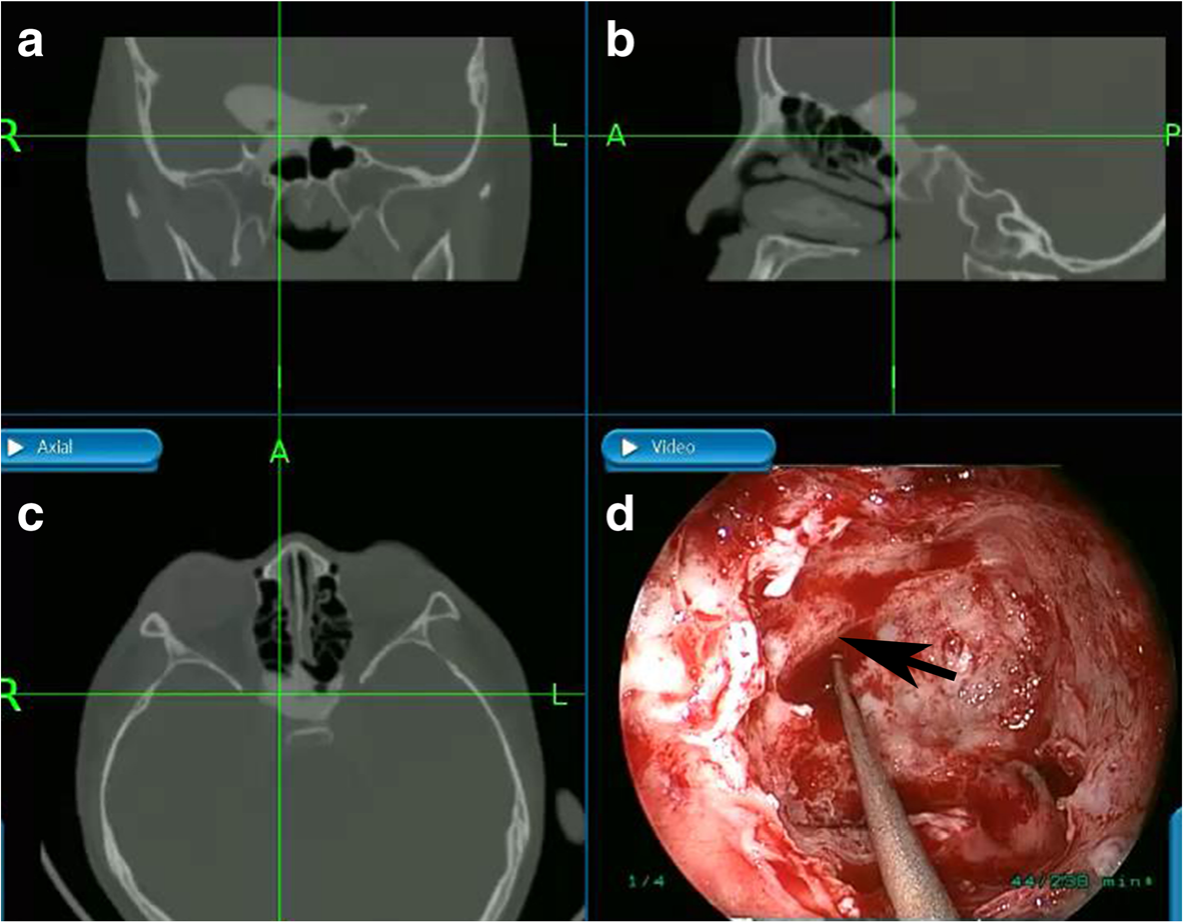
The image navigation system used on one patient with undeveloped sphenoid sinus. **a**, **b**, **c** The intersection point of the two crossing green indicates the location of optic canal. **d** Surgical image under navigation system. (The black arrow indicates the optic canal)

In order to improve the effectiveness and safety of surgery, several modifications of surgical procedures were made in our study. 1) Image navigation system was used for these pediatric patients whose sphenoid sinuses were not fully developed, to locate the optic nerve canal (Fig. 2). 2) Several pieces of cotton pad immersed with epinephrine were put into the nasal cavity before surgery to constrict the nasal mucosa, in order to reduce the hemorrhage while removing the middle turbinate. 3) Punctuated optic nerve sheath splitting was used to further decrease the pressure of optic canal. This procedure is different from the traditional way of splitting the optic nerve sheath, which is likely to cause complications like cerebrospinal fluid leakage, ophthalmic artery injury and optic nerve injury. In our procedure, 5 to 6 small intermittent punctuated incision was made to the optic nerve sheath to release the pressure with less risk of complications. In the meanwhile, splitting sheath created extra space for local administration of dexamethasone and mouse-derived NGF. The medicine accumulated in sphenoid sinus would release slowly and promote optic nerve rehabilitation continually.

## Conclusion

Children suffered from ITON with preoperative residual vision have better surgical outcome than those with NLP. For pediatric patients, time to medical treatment later than 7 days is not an independant influential factor for visual prognosis. No severe complication is encountered. Large-sampled controlled prospective studies are needed to further prove the rationality of ETOCD and to work out the exact surgical instruction.

## Abbreviation

ETOCD: Trans-ethmosphenoid optic canal decompression
HRCT: High-resolution computed tomography
ITON: Indirect traumatic optic neuropathy
NGF: Nerve growth factor RAPD Relatively afferent papillary defect
OCD: Optic canal decompression
OCF: Optic canal fracture
TON: Traumatic optic neuropathy
VA: visual acuity
VEP: Visual evoked potential
